# Metabolic Profiling Reveals Effects of Age, Sexual Development and Neutering in Plasma of Young Male Cats

**DOI:** 10.1371/journal.pone.0168144

**Published:** 2016-12-12

**Authors:** David Allaway, Matthew S. Gilham, Alison Colyer, Thomas J. Jönsson, Kelly S. Swanson, Penelope J. Morris

**Affiliations:** 1 WALTHAM Centre for Pet Nutrition, Melton Mowbray, Leicestershire, United Kingdom; 2 Metabolon Inc., Durham, North Carolina, United States of America; 3 Department of Animal Sciences, University of Illinois, Urbana, Illinois, United States of America; The University of Manchester, UNITED KINGDOM

## Abstract

Neutering is a significant risk factor for obesity in cats. The mechanisms that promote neuter-associated weight gain are not well understood but following neutering, acute changes in energy expenditure and energy consumption have been observed. Metabolic profiling (GC-MS and UHPLC-MS-MS) was used in a longitudinal study to identify changes associated with age, sexual development and neutering in male cats fed a nutritionally-complete dry diet to maintain an ideal body condition score. At eight time points, between 19 and 52 weeks of age, fasted blood samples were taken from kittens neutered at either 19 weeks of age (Early Neuter (EN), n = 8) or at 31 weeks of age (Conventional Neuter (CN), n = 7). Univariate and multivariate analyses were used to compare plasma metabolites (n = 370) from EN and CN cats. Age was the primary driver of variance in the plasma metabolome, including a developmental change independent of neuter group between 19 and 21 weeks in lysolipids and fatty acid amides. Changes associated with sexual development and its subsequent loss were also observed, with differences at some time points observed between EN and CN cats for 45 metabolites (FDR *p*<0.05). Pathway Enrichment Analysis also identified significant effects in 20 pathways, dominated by amino acid, sterol and fatty acid metabolism. Most changes were interpretable within the context of male sexual development, and changed following neutering in the CN group. Felinine metabolism in CN cats was the most significantly altered pathway, increasing during sexual development and decreasing acutely following neutering. Felinine is a testosterone-regulated, felid-specific glutathione derivative secreted in urine. Alterations in tryptophan, histidine and tocopherol metabolism observed in peripubertal cats may be to support physiological functions of glutathione following diversion of S-amino acids for urinary felinine secretion.

## Introduction

Neutering is recommended for cats by veterinarians for population welfare reasons as it reduces unwanted pregnancies, but it also has benefits for the individual, including a reduction in the risk of certain reproductive disorders and diseases [1, 2] and unwanted aggressive behaviours [3]. However, these have to be balanced against the management of some potentially undesirable consequences for the individual as neutering is a significant risk factor for obesity, which is itself associated with multiple health concerns (diabetes, dyslipidemia and osteoarthritis) [4, 5]. In cats, evidence indicates that an acute post-neuter increased food intake in *ad libitum* environments is a major driver of increased percentage body fat and body weight that persist through life and may have health consequences. Offering options to prevent weight gain associated with neutering requires an understanding of the different factors that may underpin the post-neuter dysregulation of self-regulated food intake. The importance of such data to the welfare of cats is evident when considering that the vast majority of cats (80–92%) in Europe and the US over 6 months old are neutered [6, 7].

The impact of neutering on weight is considered a consequence of two factors, a reduction in energy expenditure and increased consumption when fed *ad libitum* [8–15]. Adult cats can gain weight soon after neutering when fed an amount to maintain a pre-neuter stable body weight and it has been estimated that a reduction in intake of between 13–27% is required to maintain the pre-neuter body weight [8–10]. Similarly, post-neuter changes in energy expenditure have also been suggested in other species [11]. Evidence also exists that cats consume more if fed *ad libitum* [12, 13]. Irrespective of whether energy consumption or expenditure is the major driver of neuter-associated weight gain, weight gain is likely to be a consequence of the disruption in the cat’s endocrine milieu. Peptides regulating hunger and satiety feeding behaviours have been investigated in cats [10, 14, 15], but using different methods and in cats of different ages, under different feeding regimens and sampled over different time periods. As such, interpretation requires caution and the regulatory role of these hunger and satiety-regulating hormones in instigating any change in food intake/reduced energy expenditure has yet to be fully elucidated.

Oestrogen is a major regulator of energy intake in cats, and injection of estradiol (E2) following neutering is sufficient to prevent increased food intake and weight gain in both males and females [16, 17]. Whilst loss of oestrogen-dependent energy regulation may be the primary cause of energy imbalance following neutering in females, neutering is also a predisposition for obesity in male cats and the mechanism is less clear. If sex hormones are responsible for changing energy regulation and intake behaviour, it is possible that neutering before sexual development occurs would avoid this regulatory dominance and subsequent acute response in the post-neuter phase. Traditionally, cats are neutered around 6 to 7 months old [18], but early neutering (before or at 4 months old) is commonly performed in the US for animal welfare population control [19] and also appears to be safe [20]. However, some evidence indicate that regardless of the age at which it is performed, neutering is a significant risk factor for obesity [14, 21, 22].

Many studies investigating the effect of neutering use adult cats and allow *ad libitum* feeding. However, most cats are neutered whilst under one year old and still growing. The current NRC feeding guidelines for kittens (NRC 2006) are considered to be inappropriately high and feeding to an ideal body condition score is recommended as a more appropriate method of feeding kittens [13]. The current study aimed to establish the total energy requirements to maintain an ideal body condition score during growth up to one year old in male kittens. The impact of neutering was investigated and the study included a number of physiological measures (such as intake, body weight, body composition, spontaneous physical activity, clinical biochemistry). As kittens were fed to maintain an ideal body condition score, the opportunity for excessive weight and fat mass gain was reduced, which enabled factors that may drive acute neuter-dependent changes to be assessed (for example gut hormone, faecal microbiome and plasma metabolic profile analysis). The metabolic profiling data analysis and interpretation for male cats in that study is reported in detail here.

## Materials and Methods

### Animal maintenance and diets

The metabolic profiling study reported here refers to a cohort of 16 domestic short-haired male kittens (from 13 litters) recruited on to a trial feeding a commercially available diet, that measured food intake, body weight, body condition score, activity levels, hunger and satiety hormones, faecal microbiome, diet digestibility and metabolic profiles at different stages through to one year old. Kittens were housed in purpose-built, environmentally-enriched housing at the WALTHAM Centre for Pet Nutrition, and all housing, care and procedures were in keeping with the requirements of the Animals (Scientific Procedures) Act 1986 and the study approved by WALTHAM’s Animal Welfare and Ethical Review Body.

Kittens had free access to fresh water and were fed from a single batch of a nutritionally complete [23], commercial dry diet formulated to support kittens through growth [Royal Canin Kitten, Aimargues, France] for a minimum of 4 weeks before the first sample. Kittens were individually fed to maintain an ideal body condition score (based on the S.H.A.P.E.^™^ 7-point scale [24]) with weekly assessments to determine whether any changes in intake were required.

### Study design

Kittens were housed in a single social group and allocated to two groups based on age when they were to be neutered, with only one litter member represented in each group. Neutering was performed as part of normal veterinary practice at WALTHAM and occurred at one of two time points, defined as early (EN), at 19 weeks old and as conventional (CN), at 31 weeks old, with 8 kittens in each respective group. One kitten was removed from this study (CN group) as we were unable to obtain a blood sample in accordance with our welfare policy.

Food intake (g) was measured daily and body weight (kg) and body condition score (7-point scale) weekly. Spontaneous physical activity levels were assessed (average count for 24 hour periods over three consecutive days) using Actical devices (Philips Respironics) when cats were 19, 25, 31, 37, 43 and 52 weeks old.

### Blood sampling and peptide hormone analysis

Fasted (>16 hours) blood samples were collected from the jugular vein on up to eight occasions (at 19, 21, 25, 31, 33, 37, 43 and 52 weeks old, and prior to surgery in weeks 19 and 31). Blood (1ml) for metabolite profiling was placed in chilled EDTA tubes, mixed by inversion and incubated on ice (maximum 30 min) before centrifugation (1,000g, 10 min and 4°C). Plasma samples (~0.5ml) were collected and stored (-80°C) until transfer on dry ice to Metabolon Inc. (Durham, NC, USA), where they were stored (-80°C) until analysis.

To measure peptide hormones (insulin, ghrelin, leptin and Glucose-dependent Insulinotropic Peptide (GIP)), a Multispecies Gut Hormone Milliplex assay validated for feline samples (Merck Millipore, Watford, UK) was used according to the manufacturer’s protocol. Blood (0.5ml), collected in chilled EDTA tubes containing a DPP-IV inhibitor (5μl, cat# DPP4-010 Merck Millipore, Nottingham, UK) and protease inhibitor cocktail (5μl, cat# P2714 Sigma, Poole, UK), was centrifuged as above. Plasma was collected, snap frozen on dry ice and stored at -80°C within 30 minutes of sampling until analysis. Assays, conducted in duplicate, involved microspheres (with standards, controls and samples (25μl)), incubated at 4°C under agitation in a 96-well microtiter filter plate for 18hr. The plates were washed and detection antibody was added for 30 min at room temperature, streptavidin–phycoerythrin was then added for a further 30 min and, after washing, signal was detected on the Luminex-200 Integrated System (Luminex Corporation, Austin, TX, USA) according to manufacturer’s protocol.

### Metabolite profiling

Metabolite profiling was provided by Metabolon Inc. (Durham, NC, USA) using Ultra High Performance Liquid chromatography/Mass Spectrometry/Mass Spectrometry (UHPLC/MS/MS) and Gas chromatography/ Mass Spectrometry (GC/MS). Fractionation and derivisation of samples and detection technologies have been reported previously [25–27]. For quality assurance purposes, additional samples were included with each day’s analysis. These randomly distributed samples included extracts of a pool of well-characterized human plasma, extracts of a pool created from a small aliquot of all plasma samples (note that this also includes plasma samples from a total of 43 kittens, including females and not reported here), and process blanks. Data extraction, metabolite identification and metabolite quantification were undertaken using proprietary software.

### Data set analysis and normalisation

To enable statistical analysis in samples where metabolites were not detected, the minimum value of that metabolite that had been detected was imputed. Prior to analysis, all data were log_10_ transformed. Each individual metabolite response (S1 Table) was analysed by linear mixed effects models (LMM) with neuter status, age and their interaction as fixed effects and cat as a random effect. Models could not be fitted to six metabolites (X-12763, sucrose, atenolol, 2-oxindole-3-acetate, 12-HEPE and 1,1-kestotetraose) which were singular values (e.g. all 0) for male cats.

Planned contrasts were performed comparing the following: between neuter status, at each age; between successive time points, for each neuter status; between 19 weeks and other ages, for each neuter status and between 31 weeks and ages >31 weeks, for the conventional neuter status. The coefficients and variance-covariance matrix of each LMM were used, along with a normal approximation to the degrees of freedom, to calculate each comparison and their subsequent confidence intervals and *p*-values using simultaneous inference [28, 29]. The Benjamini–Hochberg procedure [30] was then used to adjust the *p*-values for each contrast to maintain a 5% false discovery rate (FDR) across the 364 metabolites identified.

To account for changes in intake relative to mass through growth, energy intake (calculated as kcal/kgBW^0.67^ [23]) data were also analysed by the same form of LMM as for the metabolites. Comparisons between neuter groups at each week were performed using a family wise error rate of 5%.

Statistical analyses were performed in R v 3.2.2 using the libraries *nlme* for LMM, *multcomp* for simultaneous inference and *padjust* for FDR adjustments. Means and fold changes are reported with 95% confidence intervals (CI).

Principal component analysis (PCA) was performed using the R package ‘pcaMethods’ with single value decomposition [31]. Data were log_10_ transformed and standardised prior to analysis. Ellipses were added to the PCA scores plot using the R package ‘vegan’ to illustrate 95% confidence intervals [32]. Correlation coefficient analysis was performed for both neuter groups across all time points.

### Pathway enrichment analysis

Permutation testing was then performed for each contrast to identify pathways (designated by Metabolon Inc. proprietary software) containing more significant metabolite changes than would be expected by chance. The number of metabolites in each pathway and the subset with a significant contrast were calculated. One thousand random subsets of the number of significant metabolites were then taken (to represent random significant metabolites) and the number found in each pathway calculated. The probability of a pathway containing more significant metabolite groups than would be expected by chance was calculated as the percentage of subsets where the random number in each pathway was greater or equal to the number of significant metabolites in each pathway.

## Results and Discussion

### The impact of neutering and development on physiological parameters

No significant increase in mean average daily energy intake (measured as kcal/kg body weight^0.67^) was observed in male kittens neutered at 19 weeks compared to the CN group that remained entire to 31 weeks old. However, a significant difference was observed between the two groups in weeks 34–36 and weeks 38–41 (CN group being greater, up to 36kcal/kg body weight^0.67^ with 95% CI (9, 62), S1 Fig). Epidemiological data indicated that, irrespective of neuter age, neutering is associated with increased food intake and propensity for weight gain. This study showed no intrinsic effect of neutering on energy intake relative to metabolic body weight in male cats. However, the increase in energy intake relative to metabolic body weight (compared to the EN cats) observed between weeks 3–10 post-procedure in CN cats is consistent with other post-neuter responses observed in adult male cats [15]. These data suggest that neutering males in the early stages of sexual development may reduce acute feeding behaviour changes.

Neutering is unlikely to have altered energy expenditure through changes in activity as spontaneous physical activity reduced by 25% for both groups, with no significant effect of neuter group at any time point. Peptide hormones (ghrelin, GIP, insulin and leptin) concentrations did not differ significantly between groups at any time point (data not shown), nor did faecal genera [33]. Albeit from a small cohort, these data suggest that when fed to maintain an ideal body condition score, neutering males in the early stages of sexual development has minimal impact across a spectrum of physiological functions.

### Age is the primary driver of variance in the male fasted plasma metabolome

**Fig 1:**
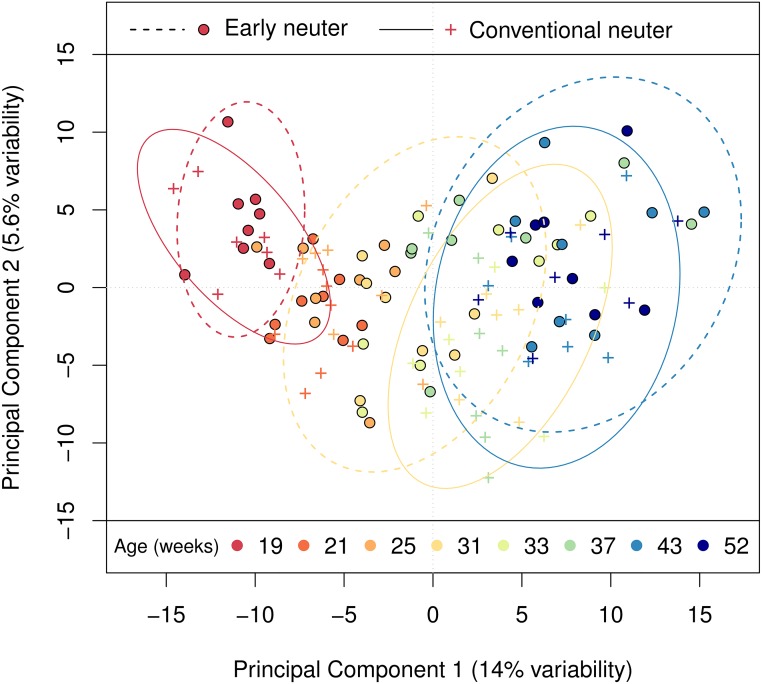
Principal Component Analysis of metabolic profiles from males indicating the impact of age on variance in the plasma metabolome. PCA of plasma metabolome samples from males, labelled by neuter group and age, indicate that age is the primary driver of variance between samples. The PCA scores plot of metabolites (with 95% confidence ellipses by neuter group at 19, 31 and 43 weeks) illustrates the divergence between groups at week 31.

Metabolic profiling detected 370 metabolites, of which 189 were consistently detectable in all samples. To determine the main drivers of variance in the plasma metabolome, multivariate analysis was performed using Principal Component Analysis (PCA) (Fig 1). Age was the primary driver of variance in the first two principal components (PC), though differences between neuter groups, observable at week 31, indicated some effect of neutering. However, the groups converged within twelve weeks of neutering the CN group, indicating that neutering age had no persistent impact on the plasma metabolome.

**Fig 2:**
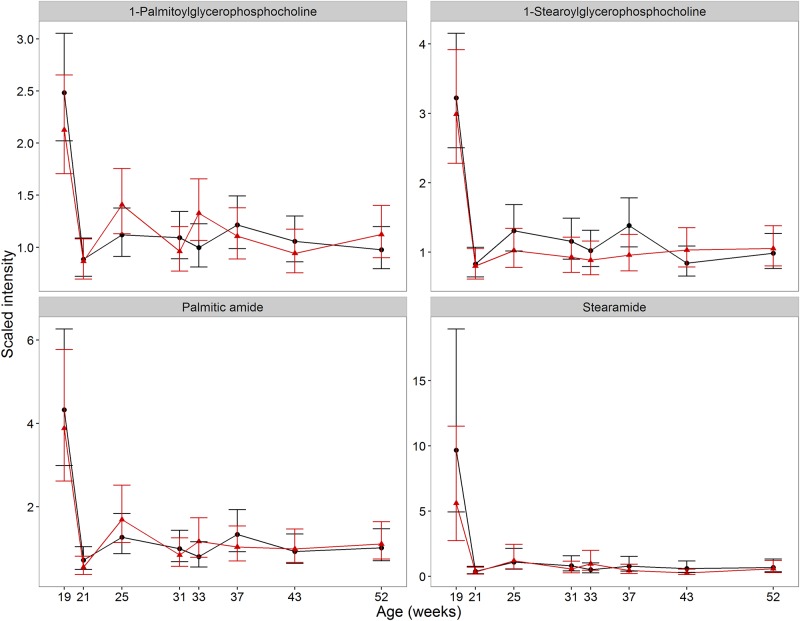
Examples of metabolites which decline between 19 and 21 weeks of age. Changes in the average abundance of metabolites for which significant changes were observed between 19 and 21 weeks of age in males in both neuter groups, CN (red) and EN (black), all of which were present in only 2 lipid metabolite subgroups (see Table 2 for details). Scaled intensity is relative to the normalised pool of all samples (error bars represent 95% CI).

Univariate analysis of metabolites that changed (FDR corrected *p*<0.05) between weeks 19 and 21 identified two groups of metabolites that decreased in both groups and remained low over the course of the study, indicative of a discrete developmental change (Table 1, with examples in Fig 2). All 4 fatty acid (FA) amides and 13 of 16 acyl glycerophosphocholines (acylGPCs) detected decreased in both groups (*p*<0.001). In mice, gene expression analysis over the pre-pubertal-to-early adult developmental phase identified changes indicative of the switch from liver growth to specialised functions, such as bile production [34]. The decline in FA amides and acylGPCs here may be due to a similar acute developmental switch and is supported by previously reported differences in lipid metabolism between kittens of 20 weeks and 32 weeks old [35].

**Table 1:** Metabolite data used to support a discrete developmental change that ended between 19 and 21 weeks old.

		Early Neuter:Age21-Age19			Conventional Neuter:Age21-Age19		
	Metabolite	Fold change	Confidence interval (95%)	FDR *p*-value	Fold change	Confidence interval (95%)	FDR *p*-value
**Fatty Acid, Amide**	**oleamide**	0.31	(0.2,0.47)	<0.0001	0.2	(0.12,0.31)	<0.0001
	**palmitic amide**	0.17	(0.1,0.27)	<0.0001	0.14	(0.08,0.24)	<0.0001
	**stearamide**	0.04	(0.02,0.1)	<0.0001	0.06	(0.02,0.16)	<0.0001
	**linoleamide (18.2n6)**	0.25	(0.17,0.37)	<0.0001	0.33	(0.21,0.51)	<0.0001
**Lysolipid**	**1-palmitoylglycerophosphocholine**	0.36	(0.27,0.48)	<0.0001	0.41	(0.3,0.55)	<0.0001
	**2-palmitoylglycerophosphocholine** *	0.56	(0.43,0.73)	0.0006	0.35	(0.26,0.46)	<0.0001
	**1-heptadecanoylglycerophosphocholine**	0.21	(0.11,0.38)	<0.0001	0.11	(0.06,0.2)	<0.0001
	**1-stearoylglycerophosphocholine**	0.26	(0.18,0.37)	<0.0001	0.27	(0.18,0.39)	<0.0001
	**2-stearoylglycerophosphocholine** *	0.3	(0.19,0.47)	<0.0001	0.13	(0.08,0.21)	<0.0001
	**1-oleoylglycerophosphocholine**	0.39	(0.29,0.52)	<0.0001	0.4	(0.3,0.55)	<0.0001
	**2-oleoylglycerophosphocholine** *	0.35	(0.24,0.5)	<0.0001	0.36	(0.25,0.53)	<0.0001
	**1-linoleoylglycerophosphocholine**	0.74	(0.62,0.88)	0.0106	0.64	(0.53,0.77)	<0.0001
	**1-eicosadienoylglycerophosphocholine** *	0.29	(0.17,0.48)	<0.0001	0.19	(0.11,0.34)	<0.0001
	**1-eicosatrienoylglycerophosphocholine** *	0.4	(0.3,0.54)	<0.0001	0.37	(0.27,0.5)	<0.0001
	**1-arachidoylglycerophosphocholine**	0.19	(0.11,0.33)	<0.0001	0.21	(0.11,0.37)	<0.0001
	**1-arachidonoylglycerophosphocholine** *	0.76	(0.65,0.89)	0.0119	0.61	(0.52,0.72)	<0.0001

Metabolites that altered significantly (FDR corrected *p*<0.05) between weeks 19 and 21 of age in both neuter groups with fold-change in means with 95% Confidence Intervals. All metabolites belonged to two groups of lipids, fatty acid amides and glycerophosphocholine lysolipids.

*Putative identification: no standard metabolite tested.

### Neutering *per se* had little acute effect on the plasma metabolome

Neutering, at any age, is reported to be associated with an increased risk of weight gain. To determine if neutering had a similar effect on metabolism irrespective of age, the metabolic profiles were analysed between the samples from the final pre-neuter time point and the first post-neuter time point two weeks later. Only retinoate and trans-4-hydroxyproline changed in both EN and CN cats (1.47- and 1.7- fold increase and 0.77- and 0.67-fold change respectively), indicating that any acute neuter-related changes were mostly resolved by two weeks post-procedure or were dominated by factors related to the different metabolic status of the groups prior to neutering.

### Metabolic differences are associated with changes in male sexual development

Univariate analysis determined metabolites differing between groups at each comparable time point (Table 2). No significant differences were observed between the groups at 19 weeks old, when both were entire, nor at 21 weeks, indicating no acute detectable effect on the metabolome 2 weeks post-neutering. Eight metabolites differed between the groups at week 25, 33 at week 31 and 19 within 2 weeks of neutering in the CN group. Only 2 metabolites were significantly different 12 weeks post-operation (week 43). These univariate analyses are consistent with PCA and indicate that despite dynamic differences as a consequence of sexual development in the CN cats, there is little evidence to suggest the age when neutered (19 and 31 weeks old) resulted in long-term effects on the plasma metabolome.

**Table 2:** Metabolites differing at some stage between neuter groups.

Ranking	Subpathway	Metabolite	19 Weeks	21 Weeks	25 weeks	31 weeks	33 weeks	37 weeks	43 weeks	52 weeks
1	**Dipeptide**	**felinylglycine** *	0.92	1.68	3.52	8.08	4.56	2.31	1.42	1.11
2	**Feline metabolism**	**gamma-glutamylfelinylglycine** *	0.91	1.47	2.94	7.24	4.04	2.1	1.4	1.06
3	**Feline metabolism**	**felinine** *	0.84	1.35	2.1	4.48	2.89	1.78	1.35	0.97
4	Tryptophan metabolism^a^	kynurenine	0.98	1.06	0.74	0.58	0.71	0.86	0.96	0.93
5	Fatty acid, dihydroxy	2-hydroxydecanoic acid	0.91	0.89	0.65	0.52	0.63	1.02	1.16	1.47
6	**Feline metabolism**	**N-acetylfelinine** *	0.93	1.3	1.77	4.64	2.92	1.91	1.22	0.98
7	**Endocannabinoid** ^a^	**N-stearoyl taurine**	1.03	1.09	2.02	3.7	4.27	3.17	2.01	2.13
8	Tryptophan metabolism^a^	tryptophan	1.08	1.12	0.97	0.74	0.85	0.94	1.05	1.03
9	Fatty acid, dicarboxylate^a^	eicosanodioate	1.16	1.17	0.91	0.61	0.72	0.96	1.05	1
10	Lysolipid^a^	1-docosahexaenoylglycerophosphocholine*	1.05	0.78	0.78	0.59	0.83	0.96	0.95	1.14
11	Cysteine, methionine, SAM, taurine metabolism	N-acetylmethionine	1	0.92	0.7	0.58	0.64	0.76	0.91	0.86
12	Pyrimidine metabolism, thymine containing	thymidine	0.85	0.92	1.63	3.24	1.65	1.07	1.17	1.18
13	Tocopherol metabolism	alpha-tocopherol	1.08	1.21	1.24	1.5	1.12	1.04	1.18	1.09
14	Sphingolipid	palmitoyl sphingomyelin	1.08	1.31	1.43	1.49	0.95	0.95	1.06	1.02
15	Histidine metabolism	histidine	0.98	0.93	0.89	0.83	0.97	0.96	0.99	1.04
16	Lysolipid^a^	1-palmitoylglycerophosphoethanolamine	0.95	0.89	0.66	0.59	0.67	1.13	1.03	0.76
17	Sterol	cholesterol	1.09	1.29	1.33	1.37	1.01	1.04	1.13	1.08
18	**Dipeptide derivative**	**anserine**	1.02	0.96	1.14	1.41	1.3	1.15	1.01	0.93
19	Lysolipid^a^	1-docosapentaenoylglycerophosphocholine*	1.32	0.79	0.79	0.56	0.96	0.96	1.24	1.09
20	**Dipeptide derivative**	**carnosine**	0.95	0.89	0.86	0.77	1.06	1.01	0.96	0.89
21	Valine, leucine and isoleucine metabolism	2-methylbutyrylcarnitine (C5)	0.97	1.11	0.86	0.62	0.81	0.98	1.08	1.11
22	Lysolipid^a^	1-oleoylglycerophosphoethanolamine	0.87	1.23	1.11	2.48	0.8	1.28	1.5	0.81
23	Alanine and aspartate metabolism	N-acetylaspartate (NAA)	2.02	1.22	1.71	3.57	1.85	1.13	1.03	1.63
24	Chemical^a^	2-ethylhexanoate	1.03	1.53	1.57	1.87	0.91	1.26	1.14	1.1
25	Glycerolipid metabolism	glycerophosphorylcholine (GPC)	1.02	1.14	1.1	1.36	0.93	0.92	1.05	0.89
26	Long chain fatty acid^a^	cis-vaccenate (18:1n7)	1.02	1.18	1.21	1.44	1.09	1.06	1.22	1.21
27	Urea cycle; arginine-, proline-, metabolism^a^	citrulline	1.04	1.07	0.77	0.66	0.78	0.92	0.88	1.05
28	Essential fatty acid^a^	eicosapentaenoate (EPA; 20:5n3)	1.04	0.92	0.88	0.69	0.8	1.04	1.01	1.32
29	Pyrimidine metabolism, cytidine containing	5-methylcytidine	1	1.02	0.9	0.71	0.68	0.82	0.98	0.79
30	Benzoate metabolism^a^	2-aminobutyrate	1.11	1.06	0.95	0.73	0.83	1.06	1.11	0.82
31	Benzoate metabolism^a^	4-vinylphenol sulfate	0.62	0.83	1.13	2.2	0.85	0.67	0.9	1.02
32	Glutathione metabolism^a^	5-oxoproline	1.02	1.04	1.01	0.83	0.88	0.93	1.02	1.02
33	Tryptophan metabolism^a^	indolepropionate	0.8	1.05	0.77	0.51	0.5	0.65	0.98	1.02
34	Essential fatty acid^a^	docosahexaenoate (DHA) 22.6n3.	0.94	0.96	0.96	0.79	0.76	0.98	0.85	0.98
35	Fructose, mannose, galactose, starch, and sucrose metabolism^a^	mannose	1.04	1.18	1.39	1.24	1.01	1.13	1.14	1.07
36	Pyrimidine metabolism, cytidine containing	2'-deoxycytidine	1	0.99	1.02	0.84	0.74	0.83	0.99	0.94
37	Creatine metabolism	creatine	1.27	1.25	1.23	1.58	2.45	1.54	1.55	1.3
38	**Glycine, serine and threonine metabolism**	**N-acetylthreonine**	0.9	1.08	0.92	0.97	0.89	0.89	0.96	0.67
39	Glutathione metabolism^a^	glutathione, oxidized (GSSG)	0.88	0.9	0.96	0.79	1.77	1.34	0.9	1.06
40	**Fatty acid, monohydroxy**	**2-hydroxypalmitate**	1.08	0.96	1.06	0.87	0.73	0.82	0.77	1.1
41	Urea cycle; arginine-, proline-, metabolism^a^	trans-4-hydroxyproline	0.91	1.13	1.02	1.07	0.67	0.82	0.88	0.78
42	Vitamin A metabolism	retinoate	1.08	0.68	0.88	1.07	1.88	1.49	1.26	1.2
43	**Food component/Plant**	**thymol sulfate**	1.05	1.19	1.07	0.95	0.87	1.08	1.59	1.13
44	Purine metabolism, adenine containing^a^	N1-methyladenosine	1.07	1.07	0.88	0.89	0.92	0.89	1.36	0.92
45	**Glycine, serine and threonine metabolism**	**serine**	1.05	1.14	1.21	0.97	0.76	0.68	0.89	0.94

Fold-change values of the 45 named metabolites identified as significantly different (FDR corrected *p*<0.05) at some stage between the two neuter groups (highlighted in red, up in CN; highlighted in green, down in CN). A further 16 unknown metabolites also met this significance cut-off, with all differences between 25–37 weeks and 11 significantly different at week 31. The list is sorted by decreasing significance values at week 31, the time point with the largest number of significant differences.

Metabolites in **bold** belong to metabolic subpathways found to have more significant metabolite groups than would be expected by chance between the two neuter groups at some time point (see text).

^a^ indicates subpathways that contained more significant metabolites than would be expected by chance within at least one of the neuter groups between timepoints.

*Putative identification: no standard metabolite tested.

An objective was to detect changes in fasted plasma samples as a consequence of neutering that may implicate a fundamental change in metabolism responsible in initiating previously observed post-neuter weight gain in cats [8, 10, 13]. No substantial evidence was found to suggest that neutering *per se* causes a change in metabolic regulation. Instead, evidence indicated that the primary differentiating driver was sexual development and changes subsequent to neutering in the CN cats. For clarification, we refer to differences between the EN and CN groups between 19 and 31 weeks old as associated with sexual development, differences in CN post-neuter as associated with consequences of sexual development (CN cats), whilst differences consistent within both EN and CN groups over time were associated with age/development.

To characterise the consequences of neutering following sexual development, metabolites that changed in the CN group from 31 weeks old and subsequent sampling points were identified (S1 Table). Many of the 85 metabolites were involved in similar areas of metabolism (39 metabolic pathways), with changes predominantly related to amino acid and lipid metabolism. To gain a broader understanding of the pathways that were most affected over time within and between groups, a Pathway Set Enrichment analysis was undertaken (Table 3). These results are described below.

**Table 3:** Metabolic pathways that differ at some stage between or within neuter groups.

	Pathways that are over-represented with significant contrasts between		
	the two groups in at least one time point comparison	at least one time point within EN group	at least one time point within CN group
Dipeptide	Y		Y
Feline metabolism	Y		Y
Tryptophan metabolism			Y
Endocannabinoid	Y		
Fatty acid, dicarboxylate			Y
Lysolipid		Y	Y
Dipeptide derivative	Y	Y	Y
Chemical		Y	Y
Long chain fatty acid		Y	Y
Urea cycle; arginine-, proline-, metabolism		Y	Y
Essential fatty acid		Y	Y
Benzoate metabolism			Y
Fatty acid, amide		Y	Y
Glutathione metabolism			Y
Glycine, serine and threonine metabolism	Y		Y
Fatty acid, monohydroxy	Y		
Food component/Plant	Y	Y	
Purine metabolism, adenine containing	Y		
Fatty acid metabolism^a^			Y
Krebs cycle			Y

Twenty pathways were found to contain more significant metabolite groups than would be expected by chance, for contrasts between groups and within groups, ranked to be consistent with the metabolites in Table 2.

^a^Pathways for which no metabolite met the univariate significant criterion used between the two neuter groups.

### Felinine metabolism and associated metabolites

**Fig 3:**
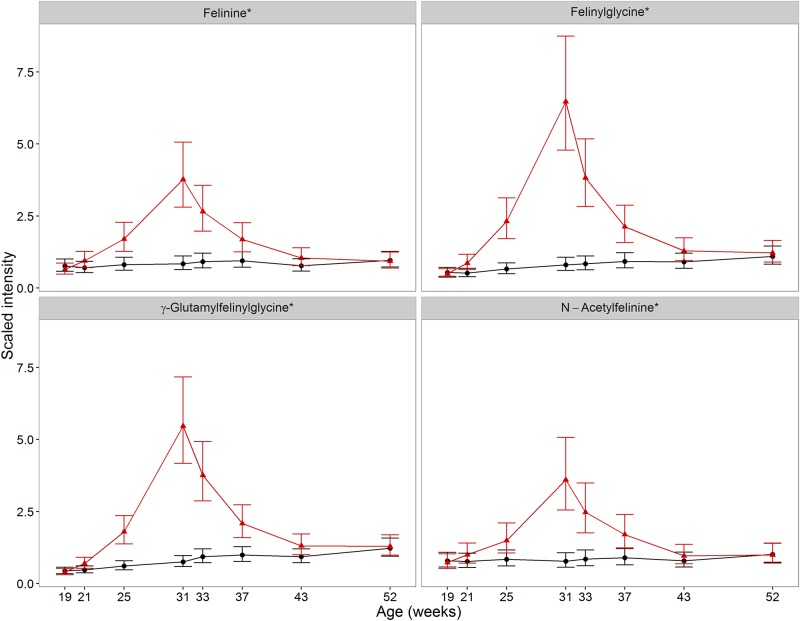
Impact of development and neutering on felinine-associated metabolites. Changes in the average abundance of metabolites of the felinine pathway and a related dipeptide in the two groups (CN (red) and EN (black)). Scaled intensity is relative to the normalised pool of all samples (error bars represent 95% confidence intervals). *Putative identification: no standard metabolite tested. These four metabolites were highly correlated (r>0.95) in the CN group of males cats.

All detected metabolites of felinine metabolism were significantly affected in CN cats (FDR corrected *p*<0.05) (Table 2, ranking (R) R2, R3 & R5), as was the dipeptide felinylglycine (Table 2, R1). These all increased in sexually maturing cats, and following neutering they decreased, until similar to EN cats by 12 weeks post-neuter (Fig 3a–3d, Table 2).

Felinine is found predominantly in the urine of sexually mature male cats, and may have a role in territorial marking and conspecific recognition [36]. Whilst there is some debate regarding the synthesis pathway of felinine itself, it is likely that the felinine precursor, γ-glutamylfelinylglycine (γ-GFG), derived from glutathione and the cholesterol precursor isopentenyl pyrophosphate, is produced in the liver [37]. γ-GFG is believed to be absorbed in the kidneys, where enzymatic activities involving γ-glutamyltransferases and N-acetyl transferases result in felinine and N-acetyl felinine synthesis [36]. As felinine and N-acetyl felinine were not detected in serum previously [37], their identification in this study may result from different methodologies and study design.

Urinary felinine is detected from 2.5–3 months of age and increases with age, predominantly in entire male cats [36]. Urinary felinine is regulated by testosterone, with neutered male cats producing approximately 3–5 fold less than entire males [36] and increasing urinary felinine in response to testosterone supplementation [38]. Whilst testosterone was not assayed here, the felinine-related data were consistent with previous reports where testosterone was detected by 5 months of age and neutering resulted in a parallel fall in plasma testosterone and urinary felinine [38]. The similar profiles from metabolites within the same pathway, interpretable with known physiological changes in sexual development in the cat over a prolonged period, provide confidence in interpretability of metabolite pools in fasted plasma.

### Tryptophan metabolism

**Fig 4:**
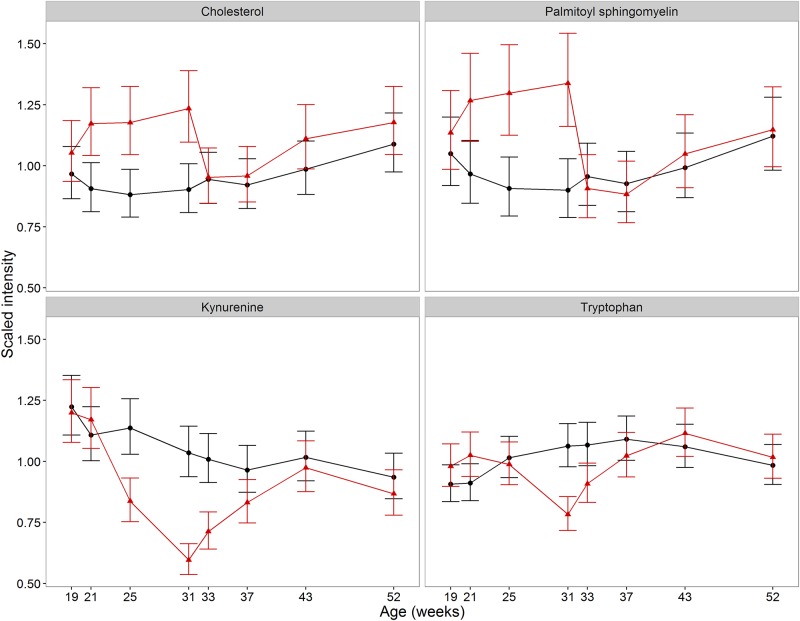
Impact of development and neutering on tryptophan and sterol metabolism. Changes in the average abundance of sterol- and tryptophan-associated metabolites with significant increases in CN compared to EN cats during sexual development ((CN (red) and EN (black)). Scaled intensity is relative to the normalised pool of all samples (error bars represent 95% CI).

Tryptophan (Table 2, R8 & Fig 4), and the tryptophan-related metabolites kynurenine (Table 2, R4 & Fig 4) and indolepropionate (Table 2, R33) were lower in EN cats compared to CN cats at week 31, (26% decrease with 95%CI (17%, 35%), 42% decrease (33%, 50%) and 51% decrease (18%, 68%) respectively). Furthermore, these and two other tryptophan-related metabolites (indoleacetate and 3-indoxyl sulfate) differed in CN cats between 31 and 43 weeks (S1 Table). These are consistent with sexual development impacting tryptophan metabolism and neutering reverting the effect. Changes in tryptophan metabolism during human adolescence have been identified [39] and in kynurenine with castration in rats [40]. The reduced pools of both tryptophan and kynurenine may indicate that synthesis of other tryptophan derivatives (such as serotonin or melatonin) are required during male sexual development (see below).

### Sterol metabolism

There is a positive association between total cholesterol and the highest testosterone:estradiol ratio in male humans [41] and testosterone replacement also positively correlates with plasma cholesterol [42]. Consistent with cholesterol’s role as a precursor for testosterone, total cholesterol (Table 2, R17; Fig 4) increased significantly in CN cats during sexual development (1.33 fold higher 95%CI (1.13, 1.57)) and declined rapidly within 2 weeks of neutering, to a similar level in EN cats. Palmitoyl sphingomyelin, a sphingolipid associated with cholesterol in human blood metabolite profiling [43], was also effected by neutering in CN cats (Table 2, R14; Fig 4) with a similar profile to cholesterol (1.49 fold higher, 95%CI (1.22, 1.81)). Sphingomyelins are especially abundant in epididymosomes, the small membranous vesicles secreted by epithelial cells within the luminal compartment of the epididymis [44]. Further research is required to determine any specific role for palmitoyl sphingomyelin in male sexual maturation/spermatogenesis in cats.

### Histidine and related dipeptides

**Fig 5:**
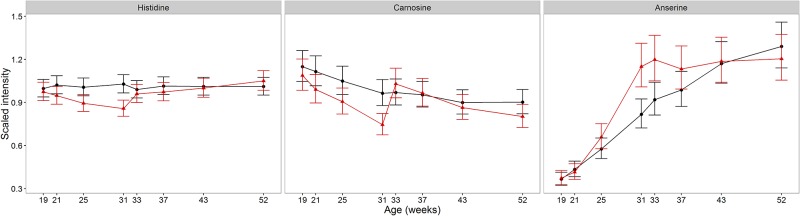
Impact of development and neutering on histidine-associated metabolites. Changes in the average abundance of histidine and histidine-derived muscle-associated amino acid derivatives. Both histidine and carnosine decrease significantly in CN compared to EN cats during sexual development and increase to levels similar to EN cats within 2 weeks of neutering (CN (red) and EN (black)). Anserine, the final product detected in this pathway increases significantly in CN cats during sexual development and remains at a stable level, whilst EN cats show a steady increase throughout development. Scaled intensity is relative to the normalised pool of all samples (error bars represent 95% CI).

Histidine, and two histidine-containing dipeptides, carnosine (β-alanyl-l-histidine) and anserine (β-alanyl-N-methylhistidine), differed between CN and EN cats at 31 weeks (Table 2, R15, R20 & R18 respectively). Compared to EN cats, histidine and carnosine decreased in CN cats during sexual development (17% lower, with 95%CI (9%, 24%) and 23% lower, with 95%CI (11%, 33%) respectively) and rapidly increased within 2 weeks post-neuter to a similar level as EN cats (Fig 5). Unlike these, the anserine pool increased during sexual development (differing to EN cats by 1.41-fold, with 95% CI (1.18, 1.69)) and remained relatively stable from 31 weeks of age. These data are consistent with upregulation of anserine synthesis for a sexual development-related requirement, with concomitant depleted pools of histidine and carnosine and, following neutering, with loss of sexual development-related anserine synthesis, resulting in carnosine and histidine being regulated to levels observed in EN males. These data are consistent with evidence of testosterone influencing muscle carnosine in mice [45, 46] and decreased muscle carnosine in adulthood, including shortly after puberty in humans [45]. As both carnosine and anserine have anti-oxidant properties [47], it is possible that the dipeptide differences seen in sexually maturing cats relate to a requirement for alternative anti-oxidants to compensate for the supply of glutathione for felinine production and secretion.

### S-amino acid derivatives, glutathione and the synthesis of alternative anti-oxidants during sexual development

**Fig 6:**
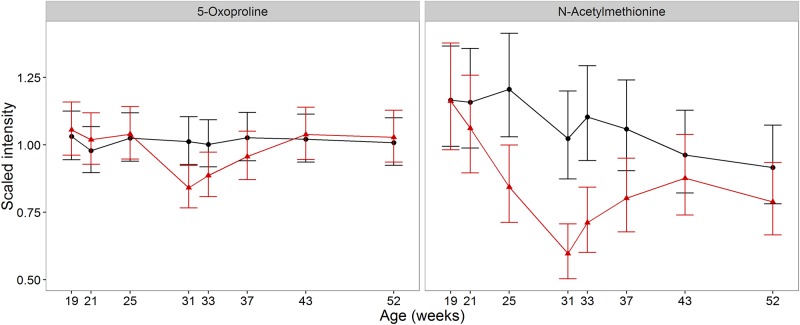
Impact of development and neutering on S-amino acid-associated metabolism. Changes in the average abundance of metabolites associated with the glutathione subpathway that differ significantly between neuter groups (CN (red) and EN (black)). Scaled intensity is relative to the normalised pool of all samples (error bars represent 95%CI).

The highly ranked metabolite N-acetylmethionine (Table 2, R11; Fig 6) is a sulphur amino acid derivative and the inverse relationship to felinine may be a consequence of partitioning sulphur amino acids toward felinine production. Whilst there was no difference in plasma glutathione (glutathione, oxidised GSSG, Table 2, R39; S2 Fig) between the groups during sexual development, at 33 weeks there was an acute increase in glutathione (1.77 fold higher, 95% CI (1.26, 2.49)) in the CN group compared with the EN group (and a 1.66-fold increase compared to CN 2 weeks previously; 95% CI (1.2, 2.3)). We propose that the glutathione pool was being maintained during sexual development, but with a greater flux driven toward felinine synthesis, with a rapid increase post-neuter following loss of felinine synthesis as glutathione synthesis outstripped demand.

Changes to other glutathione-related metabolites reflect the major impact of felinine production on S-amino acid metabolism during sexual development. 5-oxoproline (Table 2, R32; Fig 6), part of the γ-glutamyl cycle that enables glutathione-dependent uptake of amino acids into cells [48], is released by erythrocytes [49] and changes in plasma pools reflect S amino acids and glycine availability [50]. 5-Oxoproline decreased during sexual development (down 17%, 95% CI (6%, 27%) in CN cats compared to EN cats at 31 weeks) and increased following neutering (by 1.2-fold, 95% CI (1.11, 1.37) in week 43 compared to week 31 in CN, S2 Table). The changes are consistent with glutathione synthesis increasing during sexual development to support felinine production and, after neutering, the increased glutathione pool led to an increased pool of 5-oxoproline. Furthermore, glycine (S2 Table, R35; S2 Fig), a substrate for glutathione synthesis, decreased in CN cats during the acute post-neuter period.

### Other metabolites with changes associated with sexual development

**Fig 7:**
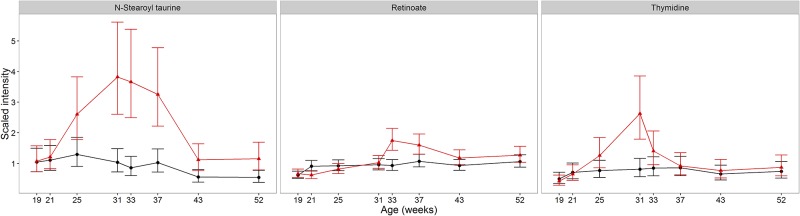
Impact of neutering on other metabolites associating with sexual development. Changes in the average abundance of metabolites associated with sexual development in male cats identified as significantly different between EN and CN cats (CN (red) and EN (black)). Scaled intensity is relative to the normalised pool of all samples (error bars represent 95%CI).

Retinoic acid regulates over 500 genes [51], drives spermatogonial differentiation and the release of spermatids from the seminiferous epithelium [52]. Whilst there was no difference between the groups during sexual development, an acute post-neuter increase in retinoate (1.7-fold, 95% CI (1.34, 2.16) between weeks 31 and 33 in CN and Fig 7) led to a difference between groups in week 33 (Table 2, R43). Similar to glutathione, this post-neuter increase suggests that retinoate synthesis and use was elevated during sexual development, and that post-neuter, an initial increase was observed before regulatory feedback resulted in levels observed in EN cats.

Metabolites showing similar profiles to felinine metabolite pools in the CN group may be similarly regulated to support sexual development. Thymidine (Table 2, R12; Fig 7) and N-acetylglycine (S2 Fig, S2 Table) were positively correlated with felinine metabolites. As thymidine is required for DNA synthesis, the increase may represent greater availability for DNA synthesis during spermatogenesis. Other nucleotide derivatives were also statistically different between neuter groups (Table 2 R30; R37). There is no report to our knowledge as to the possible role for N-acetylglycine in sexual development but as a product of glycine N-acyltransferase activity it may reflect an increased role for this phase II detoxification enzyme during sexual development, when glutathione production is supporting felinine synthesis. Furthermore, other N-acetylamino acids differed significantly during sexual development supporting the proposed increased NAT activity, regulated by testosterone (examples in S2 Fig, N-acetylaspartate (Table 2, R23); N-acetylyglycine (S2 Table).

Testosterone enhances N-acetyl transferase (NAT) activity, upregulating melatonin production in the Harderian gland of Syrian hamsters, with castrated males having similar NAT activity to females and testosterone implants in castrated males restoring NAT activty [53]. Melatonin is a highly effective antioxidant [54] and also enhances the rate-limiting step in glutathione synthesis, γ-glutamylcysteine synthase [55]. Recent evidence indicates that decreased levels of melatonin and increased levels of advanced oxidation protein products in seminal plasma are associated with human male infertility [56]. The reductions in tryptophan and kynurenine observed here may be a consequence of a testosterone-regulated increase in melatonin to protect sperm viability, providing valuable anti-oxidant protection whilst supporting glutathione production for felinine production. Therefore, it is worth noting that anti-oxidant α-tocopherol (Table 2, R14; S2 Fig) also increased through sexual development and declined rapidly following neutering. This may indicate that α-tocopherol is raised to maintain plasma anti-oxidant function whilst glutathione is directed toward felinine synthesis.

The endocannabinoid N-stearoyl taurine differed between EN and CN cats from weeks 31 to 37 (Table 2, R7, Fig 7), increasing through sexual development up to a 4-fold increase relative to EN cats in week 33. Unlike other metabolites that increased with sexual development, N-stearoyl taurine declined slower and dropped between week 37 and 42 (fold change of 0.34, 95%CI (0.22, 0.53)). This may indicate a secondary response to loss of sexual development. Little is known of the physiological role of N-acyltaurines but it has been speculated that they may function as endocrine-like signalling molecules [57].

## Summary

Previously, we have characterised factors (such as breed, gender, the individual, dietary supplementation and environment) that influence metabolic profiles in both cats and dogs [58–62]. Here, metabolic profiling has provided insights into the potential effect of development, neutering and age at time of neutering on metabolism in male kittens. Age was the major driver of variance in the plasma metabolome, with particularly striking developmental effects between weeks 19 and 21, when a significant proportion of FA amides and acylGCPs decreased. To our knowledge such acute changes in these metabolites with adolescence has not been reported. However, evidence consistent with changes in liver functionality in late adolescence exists and may indicate that in cats, liver maturation finishes between 19 and 21 weeks of age.

The objective was to investigate whether metabolic profiles changed due to neuter-dependent changes in energy intake, using EN cats with CN cats as a control group. Instead, the differences reflected sexual development to 31 weeks of age. After neutering at 31 weeks it was possible to compare the effect of age when neutered on metabolism. Whilst a post-procedure change in energy intake was observed in CN cats, the metabolome was dominated by changes that can be ascribed to the consequences of neutering on S-amino acid utilisation rather than energy metabolism, reflecting the dominance of sexual development and its’ loss.

As understanding the effect of neutering, rather than sexual development, had been the primary objective, testosterone assays were not performed and the process of sexual development can only be ascribed based on age and the known relationship between testosterone and felinine metabolism. Many metabolites that changed with sexual development were related to amino acid metabolism and can be contextualised within a network to support anti-oxidant status. Our interpretation of the data is that sexual development results in felinine production, considered important in territorial marking. Felinine production may not only have been selected due to the odorous characteristics of its breakdown products, but may also communicate “male mating quality/individual fitness value” through secretion of such a valuable resource [63]. S-amino acids are especially important in the synthesis of glutathione anti-oxidant and taurine, (the sole bile acid conjugate in cats), for which there is a dietary requirement. It is likely that a strong selection pressure exists to retain such an important class of compounds, so to secrete them as an honest signal [64] may require a compensation in metabolism (alterations in anti-oxidants derived from tryptophan, histidine and tocopherol metabolism) to secure sufficient functionality to maintain cat health status. The physiological compromises to maintain S-amino acid balance is supported by preliminary findings indicating that entire males of long-haired cat breeds may have reduced urinary felinine due to the increased requirement for cysteine in hair growth [65].

In summary, the major effect on the plasma metabolic profile was age. The acute effect of neutering *per se* showed little consistent effect on the plasma metabolome, whilst the impact of sexual development, and subsequent loss following neutering did. Statistically significant data were consistent with current understanding of male cat metabolism and also provided insights into changes through adolescence in the presence and absence of sexual development that may also be relevant to other species.

## Supporting Information

S1 Fig Energy intake. The weekly mean daily energy intake (kcal/kgBW^0.67^) for each neuter group, with means and 95% confidence intervals. Contrasts with family-wise *p*-values < 0.05 are denoted by *.
(TIFF)

S2 Fig Changes in the average abundance of metabolites associated with anti-oxidant functions and N-acetyltranserase activity. The two groups (CN (red) and EN (black)) are shown with scaled intensity relative to the normalised pool of all samples (error bars represent 95% CI).
(TIFF)

S1 Table Individual metabolite data. Dataset used for analysis.
(XLSX)

S2 Table Metabolites that differ post-neutering in CN cats. Metabolites with fold-changes that were significantly different to the 31 week sample in subsequent weeks for CN. The table is ranked to be consistent with the metabolites in Table 2. ^a^Pathways for which no metabolite met the univariate significant criterion used between the two neuter groups at week 31.
(XLSX)
