# Supplementary figures and images for: Metabolic Profiling Reveals Effects of Age, Sexual Development and Neutering in Plasma of Young Male Cats
Source: PLoS One. 2016 Dec 12;11(12):e0168144. doi: 10.1371/journal.pone.0168144 (PMC5152928; doi:10.1371/journal.pone.0168144)

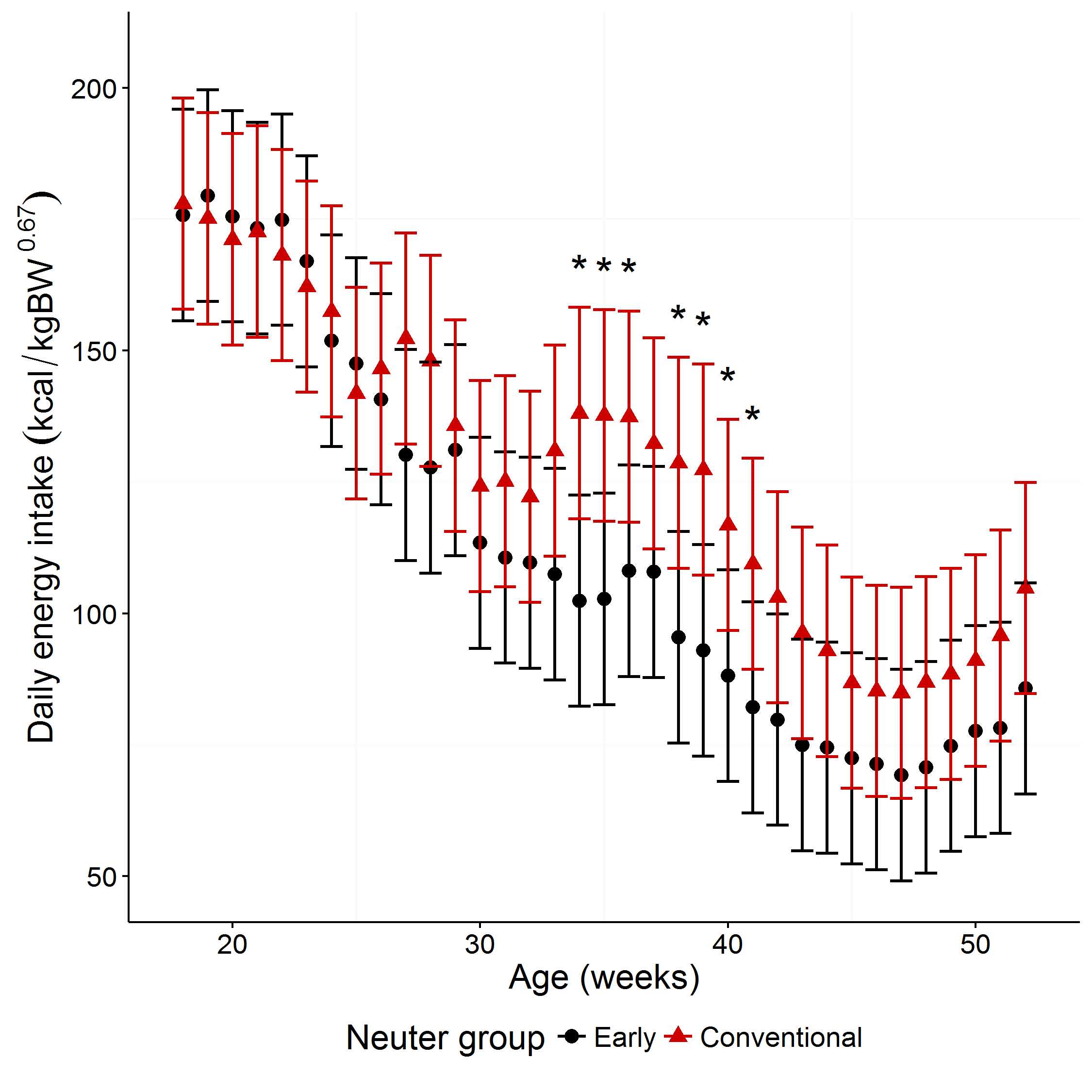

Supplement: S1 Fig — The weekly mean daily energy intake (kcal/kgBW0.67) for each neuter group, with means and 95% confidence intervals. Contrasts with family-wise p-values < 0.05 are denoted by *. (TIFF) [file pone.0168144.s001.tiff]

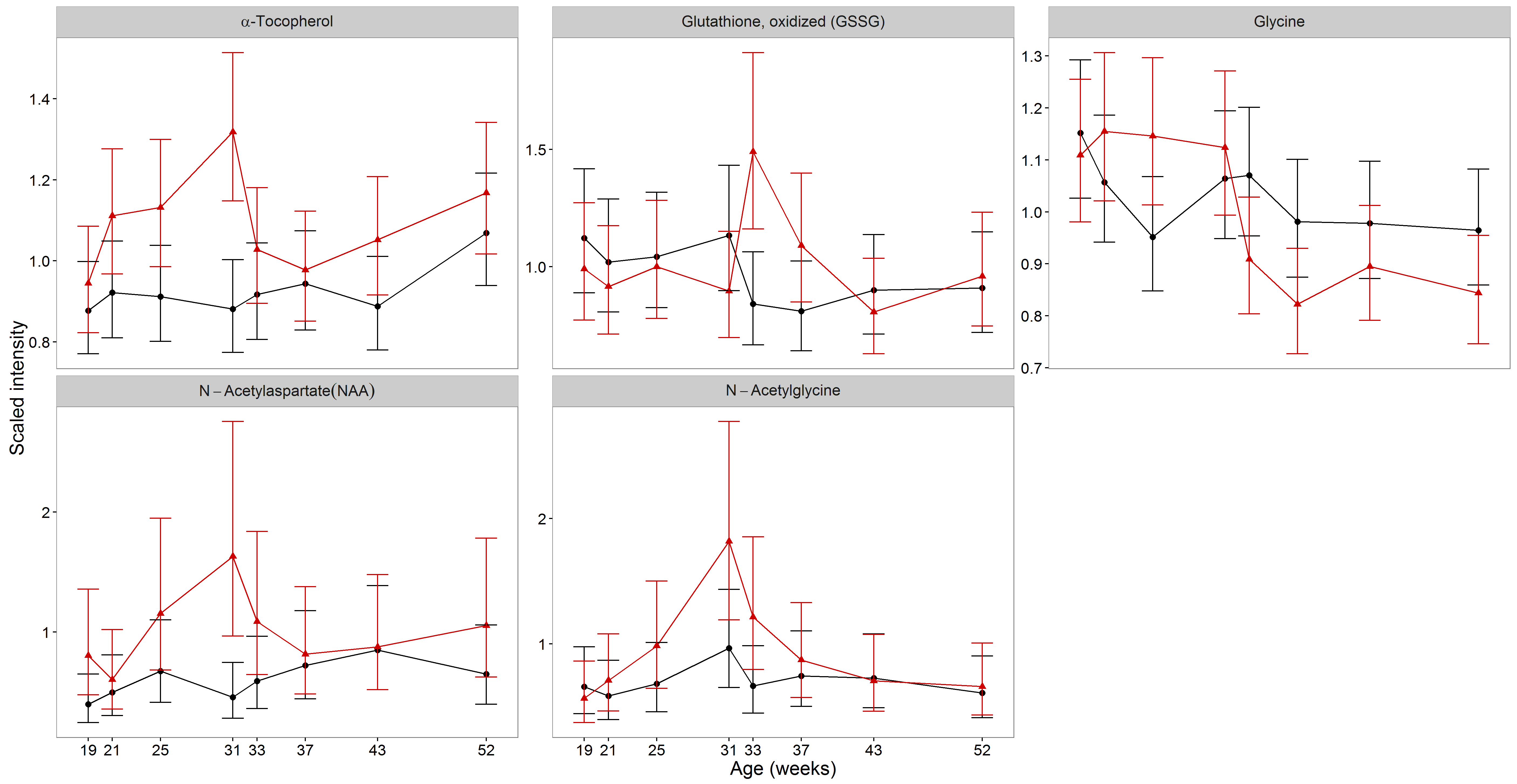

Supplement: S2 Fig — The two groups (CN (red) and EN (black)) are shown with scaled intensity relative to the normalised pool of all samples (error bars represent 95% CI). (TIFF) [file pone.0168144.s002.tiff]
